# Temporal changes in breast cancer screening coverage provided under the Brazilian National Health Service between 2008 and 2017

**DOI:** 10.1186/s12889-019-7278-z

**Published:** 2019-07-18

**Authors:** Danielle Cristina Netto Rodrigues, Ruffo Freitas-Junior, Rosemar Macedo Sousa Rahal, Rosangela da Silveira Corrêa, Pollyana Alves Gouveia, João Emílio Peixoto, Edésio Martins, Leonardo Ribeiro Soares

**Affiliations:** 1 Brazilian Breast Cancer Research Network, Advanced Center for Breast Diagnosis (CORA), School of Medicine, Federal University of Goiás, Primeira Avenida, s/n, Bloco II, Setor Universitário, Goiânia, Goiás 74605-020 Brazil; 2 Brazilian Breast Cancer Research Network, Division of Quality Control in Ionizing Radiation, National Cancer Institute (INCA), Rio de Janeiro, RJ Brazil; 3 Brazilian Breast Cancer Research Network, Faculdade Unida de Campinas, Goiânia, Goiás Brazil

**Keywords:** Breast cancer, Screening programs, Mammography, Healthcare coverage, Brazilian National Health Service

## Abstract

**Background:**

In Brazil, 70% of the population depends on the public healthcare system. Since early detection is considered crucial, this study aimed to evaluate temporal changes in breast cancer screening coverage provided under the Brazilian National Health Service (SUS) according to the different regions of the country between 2008 and 2017.

**Methods:**

This ecological study analyzed data on breast cancer screening within the SUS for women aged 50–69 years. Coverage was calculated from the ratio between the number of screening tests conducted and the expected number for the target population. Joinpoint regression analysis was used to calculate annual percent changes (APC) in coverage.

**Results:**

Around 19 million mammograms were performed in 50–69-year old women within the SUS between 2008 and 2016. The estimated APC indicates that breast cancer screening coverage increased by 14.5% annually in Brazil between 2008 and 2012 (*p* < 0.01), with figures stabilizing between 2012 and 2017 as shown by an APC of − 0.4% (*p* = 0.3). In the five geographic regions of the country, the APC initially increased, then stabilized in the north, northeast and southeast and decreased in the south and Midwest. Of the 26 states, coverage increased in seven and remained stable in six. In the other 13, there was an initial increase followed by stabilization in 11, and a reduction in coverage in two. In the Federal District, coverage remained stable throughout the study period.

**Conclusion:**

Evaluation of the temporal changes in breast cancer screening coverage provided under the Brazilian National Health Service revealed an initial increase, confirming that public policies were effective, although insufficient to ensure organized screening. There appears to be a lack of uniformity between the different regions and states and this situation is highlighted in the final 5-year period, with the APC reflecting stabilization of breast cancer screening coverage.

## Background

Several randomized clinical trials conducted between the 1960s and the 1990s have reported a reduction in mortality from breast cancer of up to 40% in the female population of 50–69 years of age submitted to breast cancer screening [1–3]. In geographical regions with limited financial resources and difficulties in accessing standard oncological treatment [4–6], this strategy may represent an opportunity for more conservative treatments and better clinical outcomes [7–9].

Despite the lack of population-based screening policy in Brazil [10–12], the disparity between access to screening within the public healthcare system, on which 70% of the Brazilian population relies, and access to screening within the private healthcare system is evident. The incidence of tumors diagnosed at advanced stages reflects this situation, since 36.9% of such cases occur in women consulting within the public healthcare network, while in the private sector this rate falls to 16.2% [13–15]. Nevertheless, this scenario has changed over recent decades, with declining mortality rates in some states of the country, possibly due to advances in the treatment of the disease [16].

Another factor that could have contributed to this stabilization was the development of programs such as the National Mammography Quality Program (PNQM) and the Cancer Database (SISCAN) [17, 18]. These initiatives aimed to improve the monitoring of the quality and reporting of test results, and patient follow-up. This represented an important step forward for public policies in the country [19, 20].

Nevertheless, there remains a need to improve such public policies. Because early diagnosis represents an important step in this process, the present study was developed to evaluate temporal changes in breast cancer screening provided under the Brazilian National Health Service (SUS) in accordance with the different regions of the country and the different states including the federal district, between 2008 and 2017.

## Methods

This was an ecological time-series study in which data referring to mammograms conducted within the SUS were analyzed, for the country as a whole, each geographic region, the states and the federal district, for the period between 2008 and 2016. Brazil consists of 26 states and a federal district, with these areas being grouped together into five geographic regions: the north, northeast, southeast, south and Midwest [21].

### Target population

The target population consisted of women of 50 to 69 years of age, according to the regulations of the Ministry of Health of Brazil [11]. Data regarding the population of women between 2008 and 2012 were collected from the System of Demographic and Socioeconomic Information on Health, Department of Information Technology of the SUS (DATASUS) [22]. The projected population of Brazil established by the Brazilian Institute of Geography and Statistics (IBGE) was used for the 2013–2016 period [23].

### Estimated coverage

Breast cancer screening coverage was estimated based on two-yearly screening aimed at reaching 100% of the target population. Coverage was expressed as a percentage and calculated from the ratio between the number of scans performed and the expected number for the target population [14].

The number of exams carried out annually between 2008 and 2017 was obtained from the DATASUS outpatient database [24] according to the codes for the procedure: 0204030030 (mammography) and 0204030188 (bilateral mammography for screening purposes). The expected number of exams for the target population was calculated from the total number of women of 50–69 years of age and in accordance with the recommendations of the National Cancer Institute (INCA) for two-yearly screening [25].

### Statistical analysis

The annual percent change (APC) in breast cancer screening coverage was calculated for Brazil as a whole, its different geographic regions, each state and the federal district. The relevant 95% confidence intervals (95%CI) were calculated, with *p*-values < 0.05 being considered statistically significant. The Poisson regression model was used for these calculations and the software program used was JoinPoint Regression, version 4.2.0.2 of June 2015 (National Cancer Institute) [26].

For analysis purposes, mammography coverage was considered to have increased when the APC increased, and the minimum value of the confidence interval was above zero. Coverage was considered to have decreased when the APC decreased, and the maximum value of the confidence interval was below zero. Coverage was considered to have remained stable when, irrespective of the rate of coverage, the minimum value of the confidence interval was below zero and the maximum value was above zero.

### Ethical aspects

The data used are publicly available [24, 25]. For this type of study, formal consent is not required. All recommendations of good clinical practice were followed according to Brazilian law and the Helsinki Convention.

## Results

In 2008, the female population of 50 to 69 years of age in Brazil was estimated at 14,432,692 women. This number increased to 19,584,342 in 2017, representing an increment of 36.0% in this population. Over that timeframe, a total of 1,227,514 and 2,790,937 mammograms, respectively, were approved for payment. This represents an increase of 127.0% in the number of exams paid for by the SUS, at a total cost of 968,567,514.42 Brazilian *reais*.

The estimated extent of breast cancer screening coverage provided under the SUS in Brazil for the 2008–2017 period ranged from 14.4 to 24.2%. Table 1 shows the estimated coverage per year for the entire study period according to the different geographic regions, the states and the federal district.

**Table 1:** Breast cancer screening coverage provided under the Brazilian National Health Service, for the country as a whole, its different geographic regions, states and the federal district for women of 50-69 years of age between 2008 and 2017

State/Region	Estimated Coverage (%)^a^									
	2008	2009	2010	2011	2012	2013	2014	2015	2016	2017
Rondônia	5.4	3.9	4.6	8.6	10.4	8.3	9.9	14.5	11.6	7.6
Acre	9.9	10.9	8.6	12.0	12.5	11.4	12.7	6.1	9.1	16.6
Amazonas	15.0	17.5	15.8	16.6	22.5	21.8	21.5	20.2	11.2	17.2
Roraima	10.2	17.0	13.2	3.4	19.4	8.2	13.4	16.0	14.5	20.1
Pará	4.6	5.8	5.9	6.4	7.1	7.5	9.8	10.2	11.5	10.6
Amapá	3.3	9.2	5.6	10.5	4.2	8.0	5.5	3.9	1.8	1.1
Tocantins	4.0	8.4	10.0	8.7	9.0	20.8	29.7	8.4	7.1	9.4
North	7.0	8.8	8.5	9.3	11.2	12.0	14.2	12.4	10.7	11.7
Maranhão	6.3	8.8	10.2	10.6	11.1	9.5	6.9	8.0	11.8	9.0
Piauí	8.5	8.9	10.8	12.6	14.5	17.4	20.0	20.3	27.5	29.6
Ceará	6.7	8.3	11.7	12.8	14.2	13.5	13.7	18.3	19.1	18.5
Rio Grande do Norte	10.8	11.8	12.5	15.7	16.5	17.3	15.7	18.6	21.5	18.3
Paraíba	9.3	10.0	8.9	10.9	14.4	20.5	26.6	21.8	22.4	21.7
Pernambuco	11.3	13.2	13.8	19.7	25.6	29.6	29.5	31.9	28.1	29.4
Alagoas	14.2	15.8	16.5	19.5	24.9	26.5	23.2	21.2	23.2	25.6
Sergipe	12.9	12.7	8.2	13.3	16.5	19.2	21.0	19.8	17.1	25.6
Bahia	21.6	18.8	16.9	20.3	32.0	29.0	32.8	30.8	37.0	34.3
Northeast	12.7	13.0	13.2	16.2	21.5	22.1	23.3	23.8	25.9	25.4
Minas Gerais	16.2	20.5	25.9	26.3	29.9	35.6	36.5	35.1	30.7	29.4
Espírito Santo	19.4	20.3	18.0	22.8	26.7	24.8	24.7	25.8	27.9	25.9
Rio de Janeiro	9.4	10.6	12.2	13.0	14.4	14.6	16.5	15.5	15.4	14.6
São Paulo	18.2	20.3	24.4	29.2	29.8	28.1	29.4	28.2	28.7	28.8
Southeast	15.9	18.2	21.8	24.7	26.3	26.8	28.1	27.0	26.3	25.8
Paraná	18.7	24.7	29.4	32.4	33.1	32.6	29.0	29.1	30.9	30.4
Santa Catarina	18.5	22.7	29.6	33.6	36.7	35.7	31.9	30.4	29.9	27.6
Rio Grande do Sul	15.2	19.2	23.3	26.4	27.5	28.1	27.1	26.9	26.4	26.2
South	17.1	21.9	26.9	30.1	31.5	31.3	28.8	28.4	28.8	28.0
Mato Grosso do Sul	9.8	13.5	16.6	17.4	20.1	18.3	21.2	19.5	20.2	18.2
Mato Grosso	7.9	7.5	10.1	12.4	11.9	11.8	11.4	10.3	10.7	8.3
Goiás	10.8	12.2	12.0	13.8	15.0	14.7	12.3	10.9	12.6	11.9
Distrito Federal	7.5	5.6	6.4	5.3	9.8	14.2	16.0	1.7	0.6	3.2
Midwest	9.5	10.3	11.5	12.7	14.4	14.7	14.4	10.5	11.3	10.6
Brazil	14.4	16.6	19.1	21.9	24.4	24.8	25.4	24.5	24.7	24.2

^a^Considering 100% of the target population

**Fig. 1:**
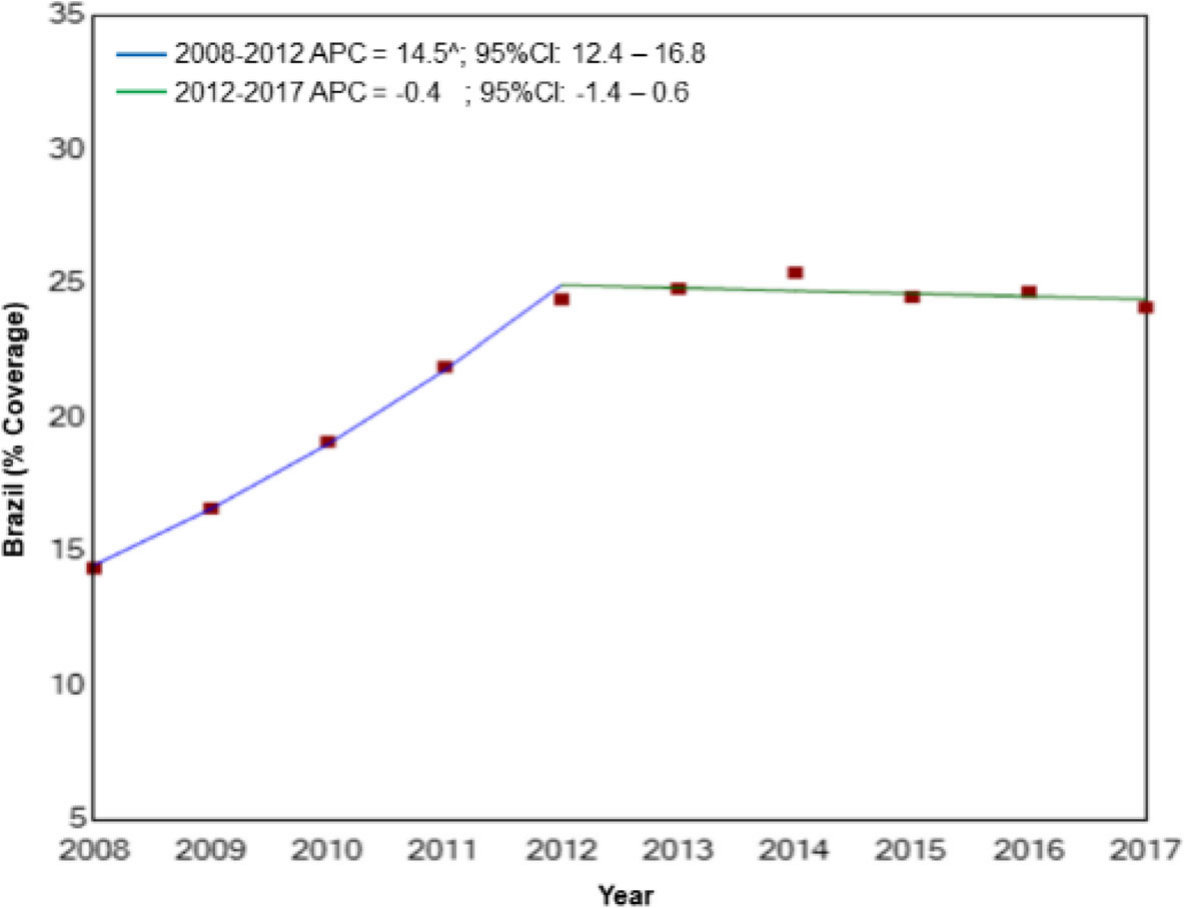
Trends in breast cancer screening coverage provided under the Brazilian National Health service for the female population of 50–69 years of age between 2008 and 2017

**Fig. 2:**
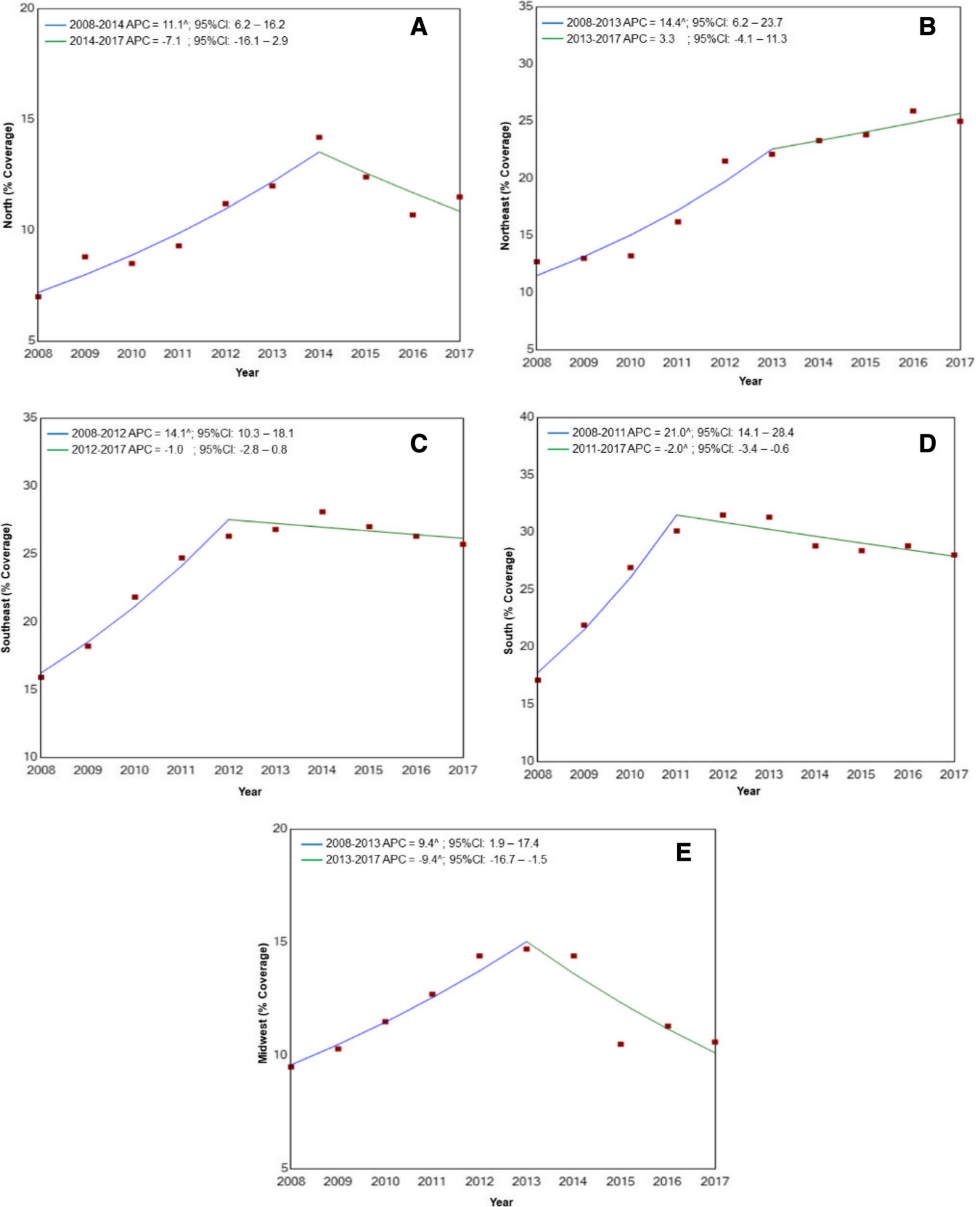
Trends in breast cancer screening coverage provided under the Brazilian National Health Service for the female population of 50–69 years of age between 2008 and 2017 according to the different geographic regions of the country. **a**) north; **b**) northeast; **c**) southeast **d**) south **e**) midwest

Regression analysis showed that, for Brazil as a whole, there was a significant increase in breast cancer screening coverage, with an APC of 14.5% (*p* < 0.01) for the 2008–2012 period, while for the 2012–2017 period coverage remained stable (*p* = 0.3) (Fig. 1). The same was true for the different regions of the country, with the analysis showing a significant increase in breast cancer screening in the north, northeast and southeast at the beginning of the study period, with APCs of 11.1, 14.4 and 14.1%, respectively (*p* < 0.01), followed by stabilization (Fig. 2a, b and c).

In the south and Midwest, however, the increase occurred between 2008 and 2011 and between 2008 and 2013, with APCs of 21.0 and 9.4%, respectively (*p* < 0.01). Nevertheless, after this period, there was a reduction in breast cancer screening coverage, with an APC of − 2.0% (*p* < 0.01) in the south and − 9.4% (*p* < 0.01) in the Midwest (Fig. 2d and e).

Of the 26 states, a significant increase in breast cancer screening coverage occurred in 7 (27%), while in 6 (23%) coverage remained stable throughout the study period. In 13 states (50%), there was significant increase in coverage at the beginning of the study period; however, after this period coverage remained stable in 11 states and decreased significantly in 2 (Table 2).

**Table 2:** Trends in breast cancer screening coverage provided under the Brazilian National Health Service in the different states and the federal district for women of 50-69 years of age between 2008 and 2017

State	Trend 1 (Years)	APC^	(95% CI)	*p* < 0.05	Interpretation	Trend 2 (Years)	APC^	(95% CI)	*p* < 0.05	Interpretation
Pará	2008–2017	10.1^	(7.5–12.7)	0.01	Increase	–	–	–	–	–
Piauí	2008–2017	15.6^	(13.9–17.3)	0.01	Increase	–	–	–	–	–
Ceará	2008–2017	9.7^	(6.3–13.3)	0.01	Increase	–	–	–	–	–
Rio Grande do Norte	2008–2017	6.3^	(3.5–9.3)	0.01	Increase	–	–	–	–	–
Sergipe	2008–2017	8.5^	(3.8–13.5)	0.01	Increase	–	–	–	–	–
Bahia	2008–2017	7.5^	(3.5–11.6)	0.01	Increase	–	–	–	–	–
Espírito Santo	2008–2017	3.8^	(1.5–6.1)	0.01	Increase	–	–	–	–	–
Acre	2008–2017	3.2	(−3.4–10.2)	0.3	Stabilization	–	–	–	–	–
Amazonas	2008–2017	0.3	(−5.3–6.3)	0.9	Stabilization	–	–	–	–	–
Roraima	2008–2017	4.0	(−4.5–13.3)	0.3	Stabilization	–	–	–	–	–
Amapá	2008–2017	−12.0	(−24.7–3.0)	0.1	Stabilization	–	–	–	–	–
Maranhão	2008–2017	1.1	(−4.0–6.5)	0.6	Stabilization	–	–	–	–	–
Rondônia	2008–2015	17.5^	(4.2–32.4)	0.01	Increase	2015–2017	−23.9	(−60.1–45.2)	0.3	Stabilization
Tocantins	2008–2014	31.8^	(10.6–57.1)	0.01	Increase	2014–2017	−36.4	(−61.2–4.1)	0.1	Stabilization
Paraíba	2008–2014	22.1^	(11.4–33.7)	0.01	Increase	2014–2017	− 4.8	(−21.0–14.7)	0.5	Stabilization
Pernambuco	2008–2013	23.7^	(14.7–33.5)	0.01	Increase	2013–2017	− 1.0	(−7.6–6.0)	0.7	Stabilization
Alagoas	2008–2012	15.9^	(2.2–31.4)	0.01	Increase	2012–2017	−0.1	(− 6.1–6.2)	1.0	Stabilization
Rio de Janeiro	2008–2014	8.9^	(6.3–11.6)	0.01	Increase	2014–2017	−4.1	(−9.6–1.8)	0.1	Stabilization
São Paulo	2008–2011	18.0^	(11.5–24.9)	0.01	Increase	2011–2017	−0.3	(− 1.7–1.1)	0.6	Stabilization
Paraná	2008–2011	18.1^	(5.4–32.4)	0.01	Increase	2011–2017	−1.9	(−4.7–1.0)	0.1	Stabilization
Rio Grande do Sul	2008–2011	20.9^	(17.2–24.7)	0.01	Increase	2011–2017	− 0.7	(−1.5–0.0)	0.1	Stabilization
Mato Grosso do Sul	2008–2012	16.3^	(3.0–31.2)	0.01	Increase	2012–2017	−1.4	(− 7.3–4.8)	0.6	Stabilization
Mato Grosso	2008–2011	21.0^	(0.7–45.5)	0.01	Increase	2011–2017	− 4.8	(− 9.5–0.2)	0.1	Stabilization
Minas Gerais	2008–2014	13.0^	(8.7–17.4)	0.01	Increase	2014–2017	−9.1^	(− 16.6 - -0.9)	0.01	Decrease
Santa Catarina	2008–2012	17.4^	(11.9–23.3)	0.01	Increase	2012–2017	− 6.4^	(−8.8 - -3.9)	0.01	Decrease
Goiás	2008–2012	7.6	(− 3.4–19.8)	0.1	Stabilization	2012–2017	−4.9	(− 10.9–1.4)	0.1	Stabilization
Distrito Federal	2008–2014	17.7	(− 1.2–40.4)	0.1	Stabilization	2014–2017	−47.7	(− 79.7–34.7)	0.1	Stabilization

^ Significance: *p* < 0.05

In the federal district, coverage remained stable between 2008 and 2014, with an APC of 17.7%. The APC for the 2014–2017 period was − 47.7% (Table 2).

## Discussion

The present study showed that there was an increase in breast cancer screening coverage provided under the SUS in Brazil between 2008 and 2017. This coverage, however, failed to reach 25% of the expected number of exams for the Brazilian population of women of 50–69 years of age. This finding confirms that the number of scans performed fails to comply with the World Health Organization recommendation that at least 70% of the target population should have access to breast cancer screening in order to effectively reduce mortality rates [27, 28].

In the past decade, the SUS has invested around 969 million *reais* in breast cancer screening, representing an increase of 92% in the number of scans performed over the period. This led to an increase in the percent coverage of the target population, from 14.4 to 24.2%, suggesting a possible improvement in the investments and public policies aimed at the early detection of breast cancer in the country [10, 18, 29].

Nevertheless, analysis of the APC for breast cancer screening coverage in Brazil showed a significant annual increase of 14.5% followed by stabilization, with a tendency towards a decrease, albeit insignificant, of − 0.4% per year from 2012 onwards. This performance differed from one geographic region of the country to another, a finding that is understandable given Brazil’s continental dimensions. Indeed, each region has different geographic characteristics and there are cultural and socioeconomic differences as well as factors inherent to income distribution, which may hamper the population’s access to healthcare services [16, 30, 31].

This poor coverage, together with the finding that the APC has stabilized, is concerning, particularly because the study population relies solely on the SUS. In fact, 70% of the Brazilian population depends exclusively on the National Health Service for access to healthcare [10, 15]. Another key point is the capacity to deliver mammography to the target population. Access depends on available appointments for screening, and geographic distance. A flattening of coverage may reflect insufficient capacity, long distances to screening facilities and lack of transportation, or no change in capacity and a large growth in population [7, 32, 33].

Recent studies have shown that mortality from breast cancer in Brazil is closely related to the human development index (HDI), with mortality rates being lower in the states with a higher HDI, while, conversely, in those with a lower HDI, the number of breast cancer-related deaths was higher [16]. A parallel can be drawn with the present study in which results show that the states in which breast cancer screening coverage provided under the SUS is highest were those with the highest HDI. These states were Minas Gerais, São Paulo, Paraná and Santa Catarina.

With respect to the geographic regions of the country, the northeast merits attention. Although breast cancer screening was below the internationally recommended level [27], an annual increase of 14.4% occurred in the first 6 years of breast cancer screening evaluated in this region. This finding may be explained by the increase in the number of mammography scanners available to the SUS and by the investment of government funding, particularly in areas such as health and education, improving human development indicators in recent years in this region [34].

On the other hand, data from the federal district, the seat of the national government, also merit attention. In the initial years analyzed in the present study, there was an increase, albeit insignificant, in breast cancer screening coverage, with an APC of 17.7% per year, which could be explained by local actions such as the inclusion of a mobile breast cancer screening program. However, problems related to the maintenance of the equipment, political instability and lack of investment in public health locally [6, 35] are factors believed to be responsible for a fall in breast cancer screening coverage, which decreased from 16% in 2014 to 0.6% in 2016, with a slight increase of 3.2% in 2017, representing an APC of − 47.7%. Although a considerable proportion of the population in the federal district has access to private healthcare, this situation may lead to an increase in diagnoses at advanced stages of the disease in the near future.

A possible under-notification of mammograms may constitute a limitation of the present study; however, this should be negligible, since the exams are only paid for after they have been included in the DATASUS platform. Nevertheless, this study illustrates the progress made in breast cancer screening coverage nationwide and may contribute towards guiding the federal government’s public policies in the control of breast cancer in Brazil, bearing in mind that the 1988 amendment to the constitution grants all citizens the right to health and establishes that the provision of healthcare is a duty of the state [36]. Therefore, all Brazilian women of 50–69 years of age have the right to a mammogram every 2 years.

## Conclusion

Analysis of temporal changes in breast cancer screening coverage provided under the Brazilian National Health Service (SUS) showed an initial increase in coverage, confirming the effectiveness of public policies. However, these were insufficient to assure an organized screening program. There was a lack of uniformity among the different regions and states, and this situation is worsening, as highlighted by the annual percent change showing that breast cancer screening coverage remained stagnant in the final 5-year period of the study.

## Data Availability

The public access to the databases is open. Links and references to databases used in the study: -DATASUS/TABNET: http://tabnet.datasus.gov.br/cgi/deftohtm.exe?ibge/cnv/popuf.def -IBGE: https://ww2.ibge.gov.br/home/estatistica/populacao/projecao_da_populacao/2013/default.shtm -SIA/DATASUS: http://tabnet.datasus.gov.br/cgi/deftohtm.exe?sia/cnv/qbuf.def -INCA: http://bvsms.saude.gov.br/bvs/publicacoes/parametros_rastreamento_cancer_mama.pdf.

## Abbreviations

APC: Annual percent changes
DATASUS: System of Demographic and Socioeconomic Information on Health, Department of Information Technology of the SUS
HDI: Human development index
IBGE: Brazilian Institute of Geography and Statistics
INCA: National Cancer Institute
PNQM: National Mammography Quality Program
SISCAN: Cancer Database
SUS: *Sistema Único de Saúde*, Brazilian National Health Service
